# Supplementary material for: Event segmentation applications in large language model enabled automated recall assessments
Source: Commun Psychol. 2025 Dec 15;3:184. doi: 10.1038/s44271-025-00359-7 (PMC12705437; doi:10.1038/s44271-025-00359-7)
Supplement: Supplementary file 2 — Reporting Summary [file 44271_2025_359_MOESM2_ESM.pdf]

Corresponding author(s): *Double-anonymous peer review submissions: write DAPR and your manuscript number here instead of author names.*

Last updated by author(s): *YYYY-MM-DD*

Reporting Summary

Nature Portfolio wishes to improve the reproducibility of the work that we publish. This form provides structure for consistency and transparency in reporting. For further information on Nature Portfolio policies, see our Editorial Policies and the Editorial Policy Checklist.

Statistics

For all statistical analyses, confirm that the following items are present in the figure legend, table legend, main text, or Methods section.

n/a	Confirmed	
☐	☒	The exact sample size (n) for each experimental group/condition, given as a discrete number and unit of measurement
☐	☒	A statement on whether measurements were taken from distinct samples or whether the same sample was measured repeatedly
☐	☒	The statistical test(s) used AND whether they are one- or two-sided<br>*Only common tests should be described solely by name; describe more complex techniques in the Methods section.*
☐	☒	A description of all covariates tested
☐	☒	A description of any assumptions or corrections, such as tests of normality and adjustment for multiple comparisons
☐	☒	A full description of the statistical parameters including central tendency (e.g. means) or other basic estimates (e.g. regression coefficient) AND variation (e.g. standard deviation) or associated estimates of uncertainty (e.g. confidence intervals)
☐	☒	For null hypothesis testing, the test statistic (e.g. F, t, r) with confidence intervals, effect sizes, degrees of freedom and P value noted<br>*Give P values as exact values whenever suitable.*
☒	☐	For Bayesian analysis, information on the choice of priors and Markov chain Monte Carlo settings
☒	☐	For hierarchical and complex designs, identification of the appropriate level for tests and full reporting of outcomes
☐	☒	Estimates of effect sizes (e.g. Cohen's d, Pearson's r), indicating how they were calculated

Our web collection on statistics for biologists contains articles on many of the points above.

Software and code

Policy information about availability of computer code

Data collection	Prompt engineered event segmentation was performed using the GPT-4 and LLaMA 3.0 API using Python 3.11.5. For human data collection, the experimental design was designed using PsychoPy 2023.2.3.
Data analysis	All statistical analyses were performed using R 4.4.0. Sentence embedding calculations for the USE, LaBSE, OpenAI , and MPNet were conducted in Python 3.11.5

For manuscripts utilizing custom algorithms or software that are central to the research but not yet described in published literature, software must be made available to editors and reviewers. We strongly encourage code deposition in a community repository (e.g. GitHub). See the Nature Portfolio guidelines for submitting code & software for further information.

Data

Policy information about availability of data

All manuscripts must include a data availability statement. This statement should provide the following information, where applicable:

- Accession codes, unique identifiers, or web links for publicly available datasets
- A description of any restrictions on data availability
- For clinical datasets or third party data, please ensure that the statement adheres to our policy

Data and analytical code are available via GitHub at github.com/ryanapanela/EventRecall.

Research involving human participants, their data, or biological material

Policy information about studies with human participants or human data. See also policy information about sex, gender (identity/presentation), and sexual orientation and race, ethnicity and racism.

Reporting on sex and gender	Gender demographics were collected at the time of experimentation. A total of 27 females and 4 males performed the experiment. Gender was not included as a factor in any analyses as previous research has not produced any significant findings which suggest major difference in event boundary patterns between genders. Similarly, our sample size recruited does not allow for a rigorous assessment of gender effects in event segmentation.
Reporting on race, ethnicity, or other socially relevant groupings	Race and ethnicity demographics were collected; however, neither were included during statistical analyses. Participants represented a diverse ethnocultural background, including individuals that identified as White or Caucasian (32%), Southeast Asian (26%), South or Central Asian (16%), African (16%), and mixed or other backgrounds (10%)
Population characteristics	Recruited participants were healthy younger adults between the ages of 18 - 35. All participants were all native or strong English speakers and possessed no known history of neurological impairments.
Recruitment	Participants were recruited from the Rotman Research Institute Participant Database, through the University of Toronto SONA pool, or expressed interest after seeing an on campus advertisement.
Ethics oversight	Rotman Research Institute, Baycrest Academy for Research and Education (#23-11).

Note that full information on the approval of the study protocol must also be provided in the manuscript.

Field-specific reporting

Please select the one below that is the best fit for your research. If you are not sure, read the appropriate sections before making your selection.

☐ Life sciences ☒ Behavioural & social sciences ☐ Ecological, evolutionary & environmental sciences

For a reference copy of the document with all sections, see nature.com/documents/nr-reporting-summary-flat.pdf

Behavioural & social sciences study design

All studies must disclose on these points even when the disclosure is negative.

Study description	Quantitative experimental
Research sample	University of Toronto Undergraduates and young adults from the Greater Toronto Area<br>This study involved the recruitment of healthy younger adults. Participants recruited were between the ages of 18 and 35 and possessed no known neurological. Due to the nature of the linguistic nature of the study, participants were required to be native or strong English speakers (having learned English before age 5). Previous research has demonstrated more stochastic event boundary patterns and reduced recall ability in older populations. As we aimed to validate automated methods that align with human patterns, we determined that recruiting a healthy younger population would be best for this study.
Sampling strategy	Sampling was completely random. Participants were recruited through the database or expressed interest as per advertisements placed throughout the University of Toronto campus. Previous research has demonstrated that event boundary patterns typically converge after a sample of 12 participants. We recruited additional participants beyond this number to ensure sufficient power if data needed to be dropped due to technical error or participant withdrawl.
Data collection	Participants were tasked to read three narrative which were provided on 8 1/2 x 11 inch paper, and mark using a pencil whenever they believed an event boundary occurred. Instructions were provided on screen using PsychoPy 2023.2.3. After reading the narrative, participants were prompted to recall the narrative in as much detail as possible by speaking into an external microphone (Shure SM7B) connected to a sound card (Steinberg UR22C).
Timing	Data collection ran from August 2023 to January 2024.
Data exclusions	No complete dataset were excluded from analysis; however, for the recall analysis, two participants were removed and one block from a third participant was removed due to a technical error during recording.
Non-participation	No participants declined to participate.
Randomization	There was no specific group allocation that required randomization. Narrative presentation order was simply counterbalanced amongst participants.

Reporting for specific materials, systems and methods

We require information from authors about some types of materials, experimental systems and methods used in many studies. Here, indicate whether each material, system or method listed is relevant to your study. If you are not sure if a list item applies to your research, read the appropriate section before selecting a response.

Materials & experimental systems

n/a	Involved in the study
☒	☐ Antibodies
☒	☐ Eukaryotic cell lines
☒	☐ Palaeontology and archaeology
☒	☐ Animals and other organisms
☒	☐ Clinical data
☒	☐ Dual use research of concern
☒	☐ Plants

Methods

n/a	Involved in the study
☒	☐ ChIP-seq
☒	☐ Flow cytometry
☒	☐ MRI-based neuroimaging

Plants

Seed stocks	*Report on the source of all seed stocks or other plant material used. If applicable, state the seed stock centre and catalogue number. If plant specimens were collected from the field, describe the collection location, date and sampling procedures.*
Novel plant genotypes	*Describe the methods by which all novel plant genotypes were produced. This includes those generated by transgenic approaches, gene editing, chemical/radiation-based mutagenesis and hybridization. For transgenic lines, describe the transformation method, the number of independent lines analyzed and the generation upon which experiments were performed. For gene-edited lines, describe the editor used, the endogenous sequence targeted for editing, the targeting guide RNA sequence (if applicable) and how the editor was applied.*
Authentication	*Describe any authentication procedures for each seed stock used or novel genotype generated. Describe any experiments used to assess the effect of a mutation and, where applicable, how potential secondary effects (e.g. second site T-DNA insertions, mosiacism, off-target gene editing) were examined.*
